# Precision medicine in colorectal cancer: genomics profiling and targeted treatment

**DOI:** 10.3389/fphar.2025.1532971

**Published:** 2025-02-27

**Authors:** Adi Muradi Muhar, Adrian Joshua Velaro, Arya Tjipta Prananda, Sony Eka Nugraha, Princella Halim, Rony Abdi Syahputra

**Affiliations:** ^1^ Department of Surgery, Faculty of Medicine, Universitas Sumatera Utara, Medan, Indonesia; ^2^ Department of Pharmaceutical Biology, Universitas Sumatera Utara, Medan, Indonesia; ^3^ Department of Pharmacology, Universitas Sumatera Utara, Medan, Indonesia

**Keywords:** precision medicine, colorectal cancer, inflammation, BRAF mutations, genomics profiling

## Abstract

Precision medicine has revolutionized the treatment of colorectal cancer by enabling a personalized approach tailored to each patient’s unique genetic characteristics. Genomic profiling allows for the identification of specific mutations in genes such as KRAS, BRAF, and PIK3CA, which play a crucial role in cell signaling pathways that regulate cell proliferation, apoptosis, and differentiation. This information enables doctors to select targeted therapies that inhibit specific molecular pathways, maximizing treatment effectiveness and minimizing side effects. Precision medicine also facilitates adaptive monitoring of tumor progression, allowing for adjustments in therapy to maintain treatment effectiveness. While challenges such as high costs, limited access to genomic technology, and the need for more representative genomic data for diverse populations remain, collaboration between researchers, medical practitioners, policymakers, and the pharmaceutical industry is crucial to ensure that precision medicine becomes a standard of care accessible to all. With continued advances and support, precision medicine has the potential to improve treatment outcomes, reduce morbidity and mortality rates, and enhance the quality of life for colorectal cancer patients worldwide.

## Introduction

The precision medicine approach has become a new milestone in the development of oncology, especially in the effort to conquer colorectal cancer. The development of genomic and bioinformatics technologies has opened up great opportunities to analyze the genetic characteristics of individuals in detail, allowing the identification of specific mutations present in cancer cells (Cortés-Ciriano et al., 2022). In colorectal cancer, common mutations that are often found include the genes KRAS, BRAF, PIK3CA, and TP53 (Jauhri et al., 2017; Sclafani et al., 2020; Afrăsânie et al., 2023; Marbun et al., 2022). These mutations play an important role in cell signaling pathways that regulate cell proliferation, apoptosis, and differentiation, which are critical points in cancer progression and aggressiveness (Feng et al., 2018). By identifying the unique genomic profile of each patient, precision medicine can be directed to target specific molecular pathways involved in the cancer process. This allows for the treatment of colorectal cancer with a more effective, efficient, and potentially reduced side effects approach compared to conventional therapies. In this approach, the genomic profile of each patient is not only a diagnostic guide but also serves as the basis for the most appropriate therapy selection (Malone et al., 2020; Naito et al., 2021). For example, patients with KRAS mutations are known not to respond well to EGFR (Epidermal Growth Factor Receptor) inhibitors, which are often used in colorectal cancer therapy (Martinelli et al., 2020; Sforza et al., 2016). With this genomic information, doctors can exclude EGFR inhibitor therapy and switch to other more appropriate therapies, such as BRAF inhibitors or MEK in cases where BRAF mutations are identified. This not only increases the likelihood of treatment success but also reduces the time it takes to determine the right therapy, so patients can benefit from faster and more personalized treatment (Prins et al., 2021).

In addition, precision medicine also allows for more adaptive monitoring of therapies. Using genomic profiling, doctors can monitor changes that occur in tumors during treatment. When cancer cells show resistance to a particular therapy, genomic information can be used to modify or change treatment strategies, thus maintaining the effectiveness of therapy as the disease progresses (Ramos and Bentires-Alj, 2015; Marine et al., 2020). This adaptive approach provides greater flexibility and opportunities to maintain control over cancer in the long term, as well as improve patients' quality of life as the treatment received is more targeted and minimizes side effects (Nikolaou et al., 2018; Salzman et al., 2018). However, the application of precision medicine in the treatment of colorectal cancer also faces various challenges. The cost of undergoing comprehensive genomic profiling is still relatively high, especially in developing countries, which can be a barrier for patients to access these genomic-based treatments (Ashenden et al., 2024). In addition, big data analysis and sophisticated bioinformatics infrastructure are needed to obtain accurate and relevant data (Tripathi et al., 2016). Access to these technologies is still limited and often concentrated in large health centers, which makes precision medicine difficult to access for a wider population, especially in rural areas.

Furthermore, genetic differences in different populations are also a challenge. For example, mutations commonly identified in Western populations do not necessarily have the same frequency in Asian or African populations (Mathieson and Reich, 2017; Gurdasani et al., 2019). For this reason, further research and data collection from various ethnic groups is urgently needed to accommodate this genetic diversity and ensure that precision medicine can be applied in an inclusive manner. To support the implementation of precision medicine, strong collaboration is needed between researchers, medical personnel, the government, and the pharmaceutical industry.

Researchers play a crucial role in developing and updating genomic knowledge as well as its analysis technologies. Meanwhile, medical personnel need to continue to update their insights in order to integrate the results of genomic analysis into daily clinical practice. The government, through supportive policies, also has a major role to play in facilitating access to this technology through subsidies, health infrastructure development, and regulations that support clinical research. On the other hand, the pharmaceutical industry needs to be involved in the development of drugs that are specific to the molecular targets found in colorectal cancers. This collaboration between stakeholders will be crucial in ensuring that precision medicine is widely accessible and able to have a significant impact in reducing morbidity and mortality due to colorectal cancer (Traversi et al., 2021; Khoury et al., 2022).

Overall, genomic profiling-based precision medicine offers a new paradigm in the treatment of colorectal cancer. By integrating genomic analysis, cancer therapies can be tailored to each patient’s unique characteristics, providing opportunities for more personalized and effective treatment. This approach not only offers hope to improve the effectiveness of treatment but also serves as the basis for the development of more advanced oncology therapies in the future. Despite still facing challenges in terms of cost, infrastructure, and accessibility, precision medicine is a promising step to reduce the incidence and mortality of colorectal cancer, as well as improve the quality of life of patients around the world.

## Genomic profile in colorectal cancer

**FIGURE 1:**
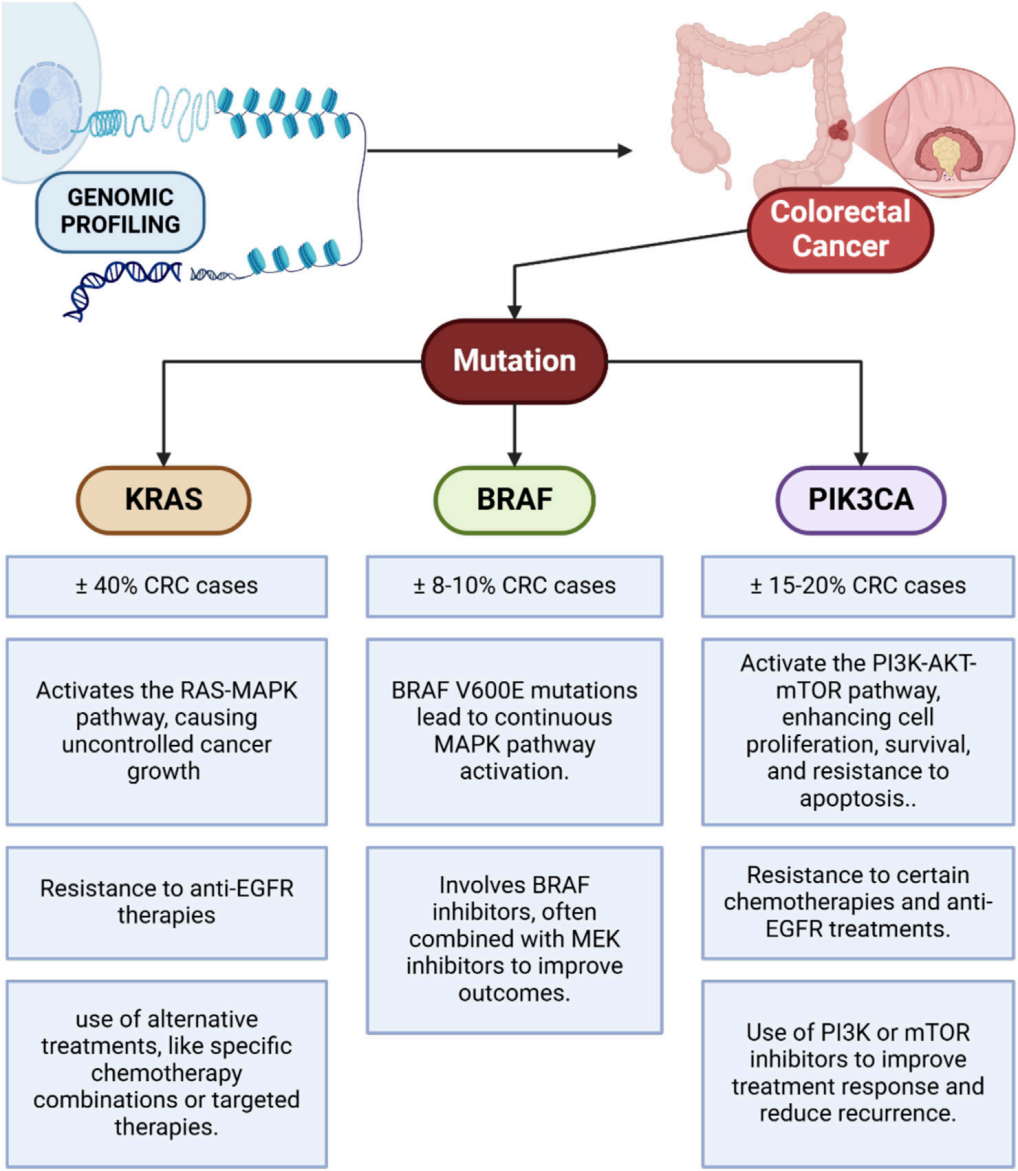
Identification of KRAS, BRAF, and PIK3CA mutations in colorectal cancer and their impact on cell signaling pathways and treatment strategies.

Genomic profiling has become an important component in the treatment of colorectal cancer, allowing for a more precise approach to understanding and treating the disease (Dalal et al., 2020; Andrei et al., 2022). Through genomic technology, the identification of specific mutations in genes associated with the development of colorectal cancer, such as KRAS, BRAF, and PIK3CA, can be performed (Figure 1). Each of these genes has a significant role in cell signaling pathways that regulate the growth, proliferation, and survival of cancer cells. Mutations in these genes can have different impacts on cancer cells’ response to therapy, greatly influencing the selection of effective treatment strategies. For example, KRAS mutations are found in about 40% of colorectal cancer cases and directly affect the RAS-MAPK signaling pathway, which is responsible for controlling cell growth and division (Ahmad et al., 2021; Vitiello et al., 2019). These mutations cause the pathway to remain constitutively active, which in turn leads to uncontrolled proliferation of cancer cells. Patients with KRAS mutations generally do not respond to anti-EGFR (Epidermal Growth Factor Receptor) therapies, such as cetuximab and panitumumab, which work by inhibiting the EGFR pathway (Li et al., 2020; García-Foncillas et al., 2019; Braig et al., 2015). Therefore, information about KRAS mutations in the patient’s genomic profile allows doctors to avoid the use of anti-EGFR therapy, which is ineffective in these cases, and look for more appropriate therapeutic alternatives, such as chemotherapy combinations with other more specific target agents.

In addition to KRAS, mutations in BRAF also play an important role in colorectal cancer, although they are less common compared to KRAS mutations. BRAF V600E mutations, for example, occur in about 8%–10% of colorectal cancer patients and are usually associated with a poorer prognosis and higher levels of aggressiveness (Bond and Whitehall, 2018; Grothey et al., 2021). These mutations cause constitutive activation of the MAPK pathway, thus allowing for the growth of cancer cells that are more aggressive and resistant to some standard treatments. In patients with BRAF mutations, BRAF inhibitors, such as vemurafenib or dabrafenib, can be used to target these mutation pathways specifically (Dankner et al., 2018). The combination use of BRAF inhibitors with MEK inhibitors has also shown better results in inhibiting this pathway (Sanchez et al., 2018), providing a therapy option that is better tailored to the patient’s needs. Meanwhile, PIK3CA mutations contribute to the activation of the PI3K-AKT-mTOR pathway, which plays a role in proliferation, cell survival, and resistance to apoptosis (Hu et al., 2021; Rascio et al., 2021). PIK3CA mutations occur in about 15%–20% of colorectal cancer cases and are often associated with resistance to some types of chemotherapy and anti-EGFR therapy (Voutsadakis, 2021; Voutsadakis, 2023). By knowing the presence of these mutations, doctors may consider the use of PI3K inhibitors or mTOR as part of therapies directed specifically at these pathways, which may improve the patient’s response to treatment and reduce the risk of recurrence.

Overall, genomic profiling provides a deeper understanding of the specific molecular characteristics that drive the development of colorectal cancer. By knowing and analyzing relevant genetic mutations, doctors can tailor therapy individually to each patient, choose the most effective drugs and combination therapies, and avoid inappropriate treatments that may not provide benefits. This not only improves the effectiveness of treatment, but also reduces the potential for side effects, improves the patient’s quality of life, and opens up opportunities for adaptive monitoring of tumor progression (Fountzilas et al., 2022; Wang and Wang, 2023). Along with technological advances and research in the field of genomics, it is hoped that precision medicine can continue to develop and become the standard in the treatment of colorectal cancer, providing new hope for patients and encouraging better treatment outcomes.

## Targeted treatment based on genomic profile

Targeted treatment in colorectal cancer is a highly specific and molecular-based approach, designed to inhibit pathways or molecules that directly trigger cancer growth and development (Rastin et al., 2024; Frank et al., 2021; Yang et al., 2022). In the context of colorectal cancer treatment, this therapy relies heavily on identifying the patient’s genomic profile, allowing for more targeted and personalized treatment. Through this approach, the therapy is not only directed at cancer cells in general but also at specific mutations or pathways found in the patient’s genome, thus providing a higher potential for treatment success compared to conventional therapies (Dietel et al., 2015). One of the main targets in the treatment of colorectal cancer is the KRAS mutation, which is found in about 40% of patients. This mutation leads to the constitutive activation of the RAS-MAPK signaling pathway, an important pathway that regulates the process of cell proliferation and division (Ahmad et al., 2021; Vitiello et al., 2019). This overactivation allows cancer cells to grow and divide uncontrollably, ultimately worsening the development of cancer. Anti-EGFR therapy, such as cetuximab or panitumumab, is commonly used to target EGFR receptors on the surface of cancer cells but is not effective in patients with KRAS mutations (Janani et al., 2022; Kasi et al., 2023). Patients with KRAS mutations tend to show resistance to this therapy, as the RAS-MAPK signaling pathway remains active even when EGFR is inhibited (Adachi et al., 2021; Chen et al., 2021). Therefore, by knowing the presence of KRAS mutations through genomic profiling, doctors can immediately avoid the use of EGFR inhibitor therapy in these patients, thereby saving time and reducing the possibility of side effects of ineffective therapy.

Alternatively, patients with KRAS mutations may be given a combination of chemotherapy or therapies that target other more relevant pathways, such as MEK inhibitors that work in the RAS-MAPK pathway (Braicu et al., 2019; Dillon et al., 2021). In addition to KRAS, BRAF mutations also play a significant role in the pathogenesis of colorectal cancers, specifically the BRAF V600E mutation, which is found in about 8%–10% of cases (Bond and Whitehall, 2018; Grothey et al., 2021). These mutations cause constitutive activation of the MAPK pathway, which promotes aggressive proliferation of cancer cells. Patients with BRAF mutations generally have a poorer prognosis and higher levels of cancer aggressiveness and tend to be resistant to some standard therapies (Florent et al., 2023). However, the development of specific inhibitors, such as vemurafenib and dabrafenib, has opened up opportunities for patients with BRAF mutations. BRAF inhibitors work by over-targeting the overactive MAPK pathway, thereby inhibiting the growth of mutated cancer cells. Studies show that the combination of BRAF inhibitors with MEK inhibitors produces better results compared to BRAF inhibitors alone (Corcoran et al., 2018; Liu et al., 2017). This combination is able to suppress MAPK pathway activity at multiple points, reduce potential resistance, and provide longer clinical response times. This suggests that genomic profile-based targeted therapy, especially for patients with BRAF mutations, can provide more precise and effective therapy options. EGFR (Epidermal Growth Factor Receptor) is also an important target in the treatment of colorectal cancer, especially in patients without KRAS or BRAF (wild-type) mutations. EGFR is a receptor that triggers signaling pathways that play a role in the proliferation of cancer cells (Santos et al., 2021). EGFR inhibitors such as cetuximab or panitumumab work by inhibiting EGFR activity on the surface of cancer cells, thereby inhibiting signaling pathways that stimulate cell growth (Cai et al., 2020). In patients with KRAS and wild-type BRAF status, EGFR inhibitor therapy has shown significant results in suppressing the progression of colorectal cancer (Ros et al., 2021; Feng et al., 2023). This confirms that EGFR inhibitors can be effective in certain groups of patients, as evidenced by the presence of genomic profiling.

However, it is important to note that although targeted therapy shows significant success, resistance can develop over time due to secondary mutations or adaptation of cancer cells to the given inhibitor (Haider et al., 2020; Labrie et al., 2022). Some patients may develop additional mutations or other resistance mechanisms that make targeted therapy less effective. For example, resistance to EGFR inhibitors may arise through the activation of alternative pathways or the development of new mutations that avoid inhibitor effects (Zhou et al., 2021; Dong et al., 2021; Cooper et al., 2022). To address this, regular monitoring of a patient’s genomic profile can help doctors identify changes in tumor characteristics and adjust therapy adaptively. Thus, this approach allows for more flexible and responsive treatment to cancer dynamics, ultimately increasing the chances of long-term treatment success.

Targeted treatment based on genomic profiles is becoming increasingly important along with technological advances and research in the field of oncology (Chakravarty and Solit, 2021; Chakraborty et al., 2024). By understanding the relevant molecular pathways and specific mutations in colorectal cancer, doctors can develop a more effective and tailored treatment plan for each patient. This approach not only increases the efficacy of the therapy but also minimizes unnecessary side effects, improves the patient’s quality of life, and reduces the risk of recurrence. Genomic profiling paves the way for a new era in colorectal cancer treatment, where therapies are no longer generic but based on the unique characteristics of each patient, bringing new hope to patients and enabling better and measurable treatment outcomes. In the future, with more and more clinical and genomic data available, targeted treatment is expected to become an integral part of colorectal cancer treatment protocols, making it the standard in oncology clinical practice. Collaboration between scientists, medical personnel, and healthcare providers is essential to ensure that this genomic profiling technology is accessible to more patients around the world.

## Benefits and challenges of precision medicine in colorectal cancer: an approach focused on genomic profiling and broader clinical implementation

**FIGURE 2:**
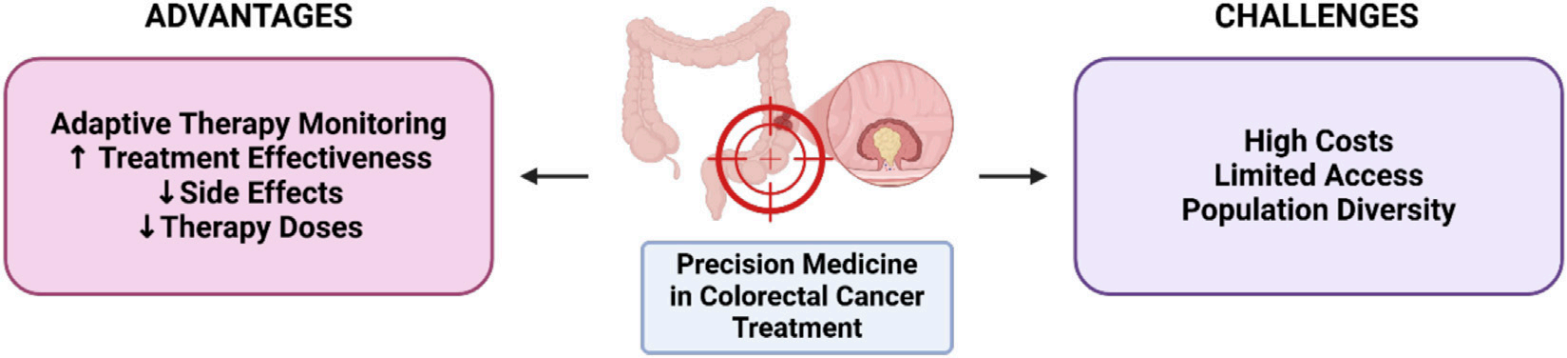
Advantages and challenges of precision medicine in colorectal cancer.

Precision medicine’s approach to colorectal cancer treatment has brought significant breakthroughs in terms of treatment effectiveness and personalization of therapy (Figure 2). By recognizing specific mutations in each patient’s genome, precision medicine provides an opportunity to target specific molecular pathways or genes that play a role in cancer cell growth, thereby maximizing the effectiveness of the therapy and minimizing side effects (Dharani and Kamaraj, 2024). In the context of colorectal cancer, this approach is very important because this cancer often involves a variety of different genetic mutations in each patient, such as mutations in the KRAS, BRAF, and PIK3CA genes (Jauhri et al., 2017; Sclafani et al., 2020). This in-depth understanding of genomic profiles allows doctors to select therapies that suit the specific characteristics of cancer cells in each patient, resulting in more precise effects and a direct impact on the success of therapy.

One of the main benefits of precision medicine is the increased effectiveness of treatment. Targeted therapies allow doctors to target mutations that trigger specific signaling pathways, such as the RAS-MAPK or PI3K-AKT pathways, that directly affect the growth and development of cancer (Yip and Papa, 2021; Karagiannakos et al., 2022). By knowing the presence of certain mutations, such as the KRAS mutation that makes patients resistant to anti-EGFR therapy, doctors can avoid the use of these ineffective therapies and switch to other, more appropriate approaches. Similarly, in patients with BRAF mutations, therapies that target those mutations, such as BRAF inhibitors and MEK, can provide better outcomes compared to conventional chemotherapy (Molina-Cerrillo et al., 2020; Djanani et al., 2020). By targeting specific molecular pathways, precision medicine enables more targeted therapies, accelerates clinical responses, and provides a higher chance of achieving positive outcomes.

In addition, precision medicine reduces the side effects that are common to conventional therapies, such as chemotherapy and radiotherapy, which attack cancer cells as well as healthy cells at the same time. Because precision medicine is designed to target specific mutations or pathways, these therapies can be delivered in lower doses and directed at cancer cells without damaging the surrounding healthy tissue (Gu et al., 2021; Bashraheel et al., 2020). This not only improves the patient’s tolerance to treatment but also reduces the risk of complications and improves the patient’s overall quality of life. Patients undergoing genomic profile-based therapy tend to experience fewer side effects, which ultimately impact treatment adherence and sustainability (Rodriguez Castells et al., 2023). The quality of life of patients is also improved because they do not have to face the severe side effects that often arise with non-specific therapies, allowing them to live a more productive and comfortable life during treatment (Faulkner et al., 2020).

Precision medicine also opens up opportunities for more adaptive therapy monitoring. As technology advances, doctors can now monitor tumor responses to therapy more dynamically and make adjustments in case of mutations or resistance changes (Edsjö et al., 2023; Ciardiello et al., 2022). For example, in cases where cancer cells develop resistance to certain inhibitors, genomic information can be used to replace or modify therapies to remain effective (Bukowski et al., 2020). This flexibility is crucial in the management of colorectal cancer, which often develops aggressively and varies from individual to individual. This adaptive monitoring also allows for early detection of new changes or mutations that may emerge over time, so that doctors can respond quickly to those changes and prevent drug resistance, which is one of the leading factors in cancer therapy failure.

However, despite these significant benefits, precision medicine in colorectal cancer also faces a number of major challenges that limit its implementation in the clinic. One of the biggest challenges is the high cost of genomic testing. The genomic profiling process requires advanced sequencing technology and detailed bioinformatics analysis, which is still relatively expensive (Arora and Tollefsbol, 2021). These technologies include state-of-the-art sequencing tools and expensive data analysis software, which makes genomic testing very expensive for most patients. In developing countries, where health budgets and financial support are limited, this price can be a major obstacle, making precision medicine accessible to only a small portion of the population (Adeniji et al., 2021; Lu et al., 2023). This condition leads to an access gap, where patients who are unable to afford it or are in a region with limited resources cannot enjoy the same benefits of precision medicine as other patients in advanced health centers. In addition to the cost problem, patient accessibility to this technology is also still very limited (Mateo et al., 2022; Ho et al., 2020). Most hospitals and health centers in various countries do not yet have the infrastructure to conduct genomic testing. These facilities are generally only available in large health centers or in developed countries, so patients in remote areas or with limited access have to travel long distances to get this test. This has an impact on delays in diagnosis and treatment for patients in areas that do not have such facilities, which ultimately affects their treatment outcomes and quality of life (Keeling et al., 2020). This accessibility issue requires serious attention, including infrastructure development and equitable allocation of resources across the region.

Population diversity is also an important challenge in precision medicine. Most of the genomic data that exists today comes from Western populations, making it less representative of Asian, African, or other ethnic groups (Atutornu et al., 2022; Cerdeña et al., 2022). Genetic variability among different ethnic groups affects the distribution of mutations and responses to therapy. The lack of data from diverse populations can lead to bias in therapy and affect the effectiveness of precision medicine if applied to other populations. For example, the frequency of KRAS or BRAF mutations in colorectal cancer may differ between different populations, so effective therapies for one population may not necessarily have the same effectiveness in another (Habashy et al., 2023; Levin-Sparenberg et al., 2020; Booker et al., 2024). Therefore, additional research and data collection from various ethnic groups is needed to expand the scope and effectiveness of precision medicine in the treatment of colorectal cancer globally.

To address these challenges, cross-sector collaboration is needed between researchers, medical professionals, governments, and the pharmaceutical industry. Researchers can play a role in developing more affordable genomic technologies and expanding representative genomic databases. Meanwhile, medical professionals need to be trained in the interpretation and application of genomic results to support the implementation of precision medicine in the clinic. The government also has a big role to play in providing subsidies and supporting infrastructure development, as well as in designing policies that support equitable access to these technologies. The pharmaceutical industry, on the other hand, needs to be involved in the development of more affordable targeted medicines and promote global accessibility. Overall, although precision medicine offers promising prospects in the treatment of colorectal cancer, its implementation still faces challenges in terms of cost, accessibility, and diversity of genomic data. However, with continued advances and support from various sectors, precision medicine has the potential to become a new standard in cancer treatment, providing better treatment opportunities and improving the quality of life of colorectal cancer patients worldwide.

## The future of precision medicine in colorectal cancer treatment: potential, challenges, and collaboration for a more effective standard of treatment

With the rapid development of technology and the increasing amount of genomic data available, the precision medicine approach has great potential to become the standard in the treatment of colorectal cancer in the future. Precision medicine allows doctors to tailor therapy based on each patient’s specific genomic profile, maximizing the effectiveness of treatment, and minimizing side effects. As one type of cancer that involves various genetic mutations such as in KRAS, BRAF, and PIK3CA, colorectal cancer greatly benefits from this genomics-based approach. In this context, the future of colorectal cancer treatment will focus on increasing the availability and accessibility of precision medicine for different populations and the development of more specific and effective therapies (Middleton et al., 2022; Bando et al., 2023).

### Improvement of genomic data and analysis technology

One of the key factors driving precision medicine towards wider implementation is the increasing availability of genomic data. Today, large-scale genomics projects, such as the Human Genome Project and the Cancer Genome Atlas project, have produced rich data on genetic variation in different types of cancer, including colorectal cancer (Ganini et al., 2021; ICGC/TCGA Pan-Cancer Analysis of Whole Genomes Consortium, 2020; Guo et al., 2021). This data includes not only the most common mutations but also minor variations that may affect a patient’s response to therapy. With more and more data available, doctors can understand more about the specific mutation profiles in colorectal cancers and develop more accurate and effective therapeutic protocols. In addition, advances in sequencing technology, such as Next-Generation Sequencing (NGS), have enabled genomic profiling at higher speeds and lower costs (Satam et al., 2023; Gupta and Gupta, 2020). This technology makes the DNA sequencing process faster and more affordable so that genomic analysis can be applied more widely. In the future, it is hoped that this technology will become more affordable and accessible, allowing precision medicine to be integrated into clinical care standards for more colorectal cancer patients. With more affordable testing costs, precision medicine can be accessed by people from all walks of life, thereby increasing equity in cancer treatment.

### Cross-sector collaboration: researchers, medical practitioners, and policymakers

For precision medicine to become the standard of care, close collaboration between researchers, medical practitioners, and policymakers is needed. Every stakeholder has a crucial role to play in realizing precision medicine that is more affordable, accessible, and has a real impact on colorectal cancer patients. Researchers in the fields of molecular biology, oncology, and bioinformatics have an important role to play in expanding the understanding of genetic mutations specific to colorectal cancer. They also need to develop more accurate and efficient algorithms for analyzing genomic data to assist doctors in making quick and precise clinical decisions (Hassan et al., 2022; Quazi, 2022). Broader and more representative research is needed to enrich genomic databases with data from various ethnic groups so that precision medicine can be applied in an inclusive manner (Tawfik et al., 2023). On the medical side, doctors and health workers need to be trained in the interpretation of genomic profile results and the application of targeted therapies in daily clinical practice. Many doctors may not have experience with precision medicine, so training and capacity building are essential. In addition, medical practitioners need to understand the importance of adaptive monitoring, where they must be prepared to modify or replace therapy if cancer cells show signs of resistance. Continuing education and expertise development in precision medicine can accelerate the integration of these approaches in clinical protocols. Policymakers have a strategic role to play in creating regulations and policies that support the development and access to precision medicine. Government subsidies for genomic testing, for example, can reduce the costs that patients have to bear, especially in developing countries. In addition, policies that encourage clinical research and the development of health infrastructure that supports precision medicine can accelerate the adoption of this approach across the region (Afzal et al., 2020). Policymakers also need to ensure that these technologies and healthcare services are available equally so that precision medicine is accessible to patients from different backgrounds and locations.

### Challenges to overcome for a more inclusive future of precision medicine

Although precision medicine offers a variety of benefits, there are still challenges that need to be overcome in order for this approach to become the standard in the treatment of colorectal cancer. In addition to the high cost, other challenges are the gap in access to technology and inequality in the availability of genomic data (Ory et al., 2023). Most of the current genomic data comes from Western populations, making it less representative of other populations, including Asian and African populations. Genetic variation between populations can affect the effectiveness of therapy, so additional research is needed to expand the scope of more inclusive genomic data (Sharif et al., 2020; Fernández-Rhodes et al., 2020). With more representative data, precision medicine can be applied more effectively and evenly around the world. In addition, to deal with potential drug resistance, precision medicine needs to develop a more responsive and adaptive monitoring system in the future (Beitler et al., 2022). Resistance to targeted therapy often develops as new mutations develop in cancer cells, which can reduce the effectiveness of treatment. Therefore, a more flexible monitoring system is needed to detect these changes early and adjust therapy according to the progression of the disease.

### The prospect of precision medicine as the future standard of medicine

With strong collaboration between researchers, medical practitioners, and policymakers, precision medicine has great potential to become the standard in colorectal cancer treatment in the future. This process may take significant time and investment, but the results will provide great benefits to patients around the world (Denicolai and Previtali, 2020). With the widespread application of genomic technology, it is hoped that precision medicine can provide more effective, safe, and personalized therapy for colorectal cancer patients, improve their quality of life, and reduce morbidity and mortality rates. The success of precision medicine depends not only on technology but also on how this approach is integrated into the global health system. Policy support, infrastructure development, and education for healthcare professionals are key elements to make precision medicine a solution that is accessible to all patients, regardless of background or geographic location. Precision medicine is a promising future in colorectal cancer treatment, providing new hope for more effective and sustainable therapies, as well as pushing patients’ quality of life to a better level.

## Conclusion

Genomic profiling-based precision medicine brings new promise in colorectal cancer treatment, enabling a more personalized and specific approach based on each patient’s genetic characteristics. With the ability to target specific genetic mutations or molecular pathways such as KRAS, BRAF, and PIK3CA, precision medicine is able to improve the effectiveness of treatment and reduce side effects, thereby significantly improving the patient’s quality of life. This approach not only focuses on more targeted treatments but also allows for adaptive monitoring of cancer cell progression, providing the flexibility to tailor therapy according to the patient’s changing condition. However, while precision medicine promises to have a major impact in treating colorectal cancer, its wider implementation still faces challenges, especially in terms of high costs, limited access to genomic technology, and the need for more representative genomic data for diverse populations. Therefore, continuous efforts are needed in terms of inclusive research, the development of more affordable technologies, and policy support from the government as well as cross-sector collaboration to expand access to precision medicine. With comprehensive support from various parties, precision medicine has the potential to become a standard of care that can be accessed by all levels of society, providing new hope for more effective and sustainable treatment of colorectal cancer.
